# Lens Transplantation in Zebrafish and its Application in the Analysis of Eye Mutants

**DOI:** 10.3791/1258

**Published:** 2009-06-01

**Authors:** Yan Zhang, Kyle McCulloch, Jarema Malicki

**Affiliations:** The Second Teaching Hospital of Jilin University; Department of Ophthalmology, Harvard Medical School

## Abstract

The lens plays an important role in the development of the optic cup^[1,2]^. Using the zebrafish as a model organism, questions regarding lens development can be addressed.  The zebrafish is useful for genetic studies due to several advantageous characteristics, including small size, high fecundity, short lifecycle, and ease of care.  Lens development occurs rapidly in zebrafish.  By 72 hpf, the zebrafish lens is functionally mature ^[3]^. Abundant genetic and molecular resources are available to support research in zebrafish.  In addition, the similarity of the zebrafish eye to those of other vertebrates provides basis for its use as an excellent animal model of human defects^[4-7]^. Several zebrafish mutants exhibit lens abnormalities, including high levels of cell death, which in some cases leads to a complete degeneration of lens tissues ^[8]^.

To determine whether lens abnormalities are due to intrinsic causes or to defective interactions with the surrounding tissues, transplantation of a mutant lens into a wild-type eye is performed. Using fire-polished metal needles, mutant or wild-type lenses are carefully dissected from the donor animal, and transferred into the host.  To distinguish wild-type and mutant tissues, a transgenic line is used as the donor.  This line expresses membrane-bound GFP in all tissues, including the lens.  This transplantation technique is an essential tool in the studies of zebrafish lens mutants.

**Figure:**
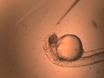


## Protocol

### Part 1:  Preparing the embryos

In this protocol, we will use the jj^xy^ symbol to denote a hypothetical zebrafish lens mutant.

Zebrafish strains are maintained in standard fish facility conditions at 28.5  C on a 14h light/10h dark cycle.
In the evening, place males and females of the zebrafish strain *jj^xy^:AB/TU*; tg(*mGFP*) in a tank with a divider to separate them from each other.  The progeny of this cross will express the GFP transgene in all tissues, and will be used as donors.  In parallel, use the same procedure to set up crosses between non-transgenic wild-type animals.  Depending on the purpose of this experiment, one can cross animals wild-type at the *jj^xy^* locus as donors or hosts.
Within one hour after the light turns on in the morning, remove the dividers to allow the fish to mate.
After 15-30 minutes collect embryos in 100 x 15 mm Petri dishes, clean them, and change the medium (egg water).
Keep the embryos in a 28.5  C incubator until desired time.
When ready for transplantation, remove the chorion manually with two pairs of sharp forceps, and incubate the embryos in 0.2%EDTA in calcium-free ZFR for 30 minutes.

### Part 2:  Preparation of dissection needles

The transplantation procedure requires two types of needles: a sharpened one, which is used to cut tissues surrounding the lens, remove the chorion, and release embryos from agarose; and a blunt needle used to move the donor lens and insert it into the host.  Here we will show you how to make these needles.
First, the tip of a Pasteur pipette must be cut off using a diamond knife. Then insert a glass capillary into the Pasteur pipette tip so that about half of its length is inside it.  This will make it easier to immobilize the tungsten wire by melting it into the glass in the next step.
Then insert a thin tungsten wire into the open end of the capillary.  Melt the glass over the wire with a Bunsen burner.  Using forceps, hold steady the glass end with the metal wire while twisting the other end of the needle with your hand.  The softened glass should spiral around the metal gripping it in place.
If you are making a blunt tip needle, then this is the end of the procedure.  To create a sharpened needle, hold the tungsten wire over a Bunsen burner for 1-1.5 minutes, burning off the metal so a very fine tip is created. Check the quality of tip by microscropy.It is a good idea to sterilize both needles in the flame for a few seconds immediately before transplantation.

### Part 3:  Preparation of reagents

Calcium-free Zebrafish Ringer’s (ZFR) Solutions (116 mM NaCl, 2.9 mM KCl, 5 mM HEPES, pH 7.2)
0.2%EDTA in calcium-free Zebrafish Ringer’s Solutions (pH 7.2)
1.2% agarose (low melting point) in calcium-free ZFR
0.2% agarose (low melting point) in calcium-free ZFR
Embryo medium (pH 7.0, per liter contains: 10 ml Hanks Solution #1, 1ml Hanks Solution #2, 10ml Hanks Solution #4, 10ml Hanks Solution #5, 0.35 g sodium bicarbonate, 300 uL of 2M HCl, penicillin-streptomycin 500,000U) As in *The Zebrafish Book* (University of Oregon Press, 2000).
Tungsten wire, gold plated, 0.1mm diameter, Alfa Aesar
Capillary tube, 1.0mm diameter, World Precision Instruments

### Part 4: Transplantation procedure

In a plastic centrifuge tube, prepare 1.2% low melting point agarose in calcium-free ZFR preheated to 40 C in a water bath.
At the desired stage (30 hpf, for example), take up the embryos in a Pasteur pipette, slowly expel them into the centrifuge tube, and slowly expel them into the centrifuge tube and incubate for about 5 seconds.  The donors and hosts should be incubated separately.
With a pipette, take up the embryos in 1.2% agarose from the centrifuge tube and arrange them in two parallel rows in a 100mm polystyrene Petri dish, keeping hosts and donors separate.  By pipetting them out slowly and carefully, most embryos will lie on their sides in the agarose.  You will need to reorient those that do not lie in this position using the blunt needle so that the eye is facing up before the agarose solidifies.
Wait for the 1.2% agarose to solidify, and then overlay it with 0.2% low melting point agarose solution.
Using the sharpened needle, remove any agarose around the eyes, and very carefully cut the lens out from the host and donor embryos using small strokes.  Make sure to cut just close enough to the lens so as not to tear it, but also so you do not remove too much tissue from the rest of the host eye.
Once free, lenses will float in the medium.  Using the blunt needle, carefully push the donor lens to just above the location of where the host lens would be normally, and then push it down into the eye with the blunt needle. The host lens may be discarded.
Leave donor and host embryos in 1.2% agarose for 30-60 minutes, then release them from the agarose using the sharp needle, and transfer them into a 24-well plate with embryo medium.  Each embryo should occupy a separate well.  Make sure to label the wells properly so that it is clear which host corresponds to which donor.
Incubate at 28.5 C. The donor-derived lens can be distinguished from the host lens on the unoperated side of the same animal based on GFP fluorescence.  Sections can also be made to measure lens size and to determine whether it differentiates properly.

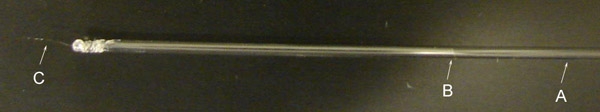
 **Fig. 1** Low magnification image of a sharpened needle used for lens transplantation. A glass capillary (B) is inserted into the Pasteur pipette (A), and a thin tungsten wire (C) is inserted into the open end of the capillary.  The glass over the wire is softened with a Bunsen burner.  Using forceps, hold steady the glass end with the metal wire while twisting the other end of the Pasteur pipette with your hand.  The softened glass should spiral around the metal, gripping it in place.


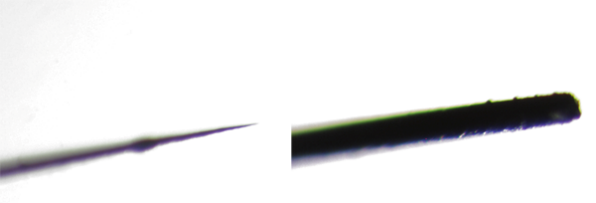
 **Fig. 2** The tips of the two kinds of needles used for lens transplantation.


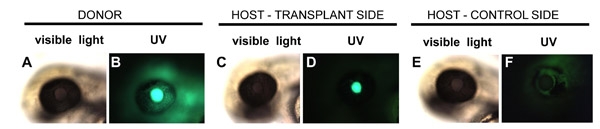
 **Fig. 3** Embryos underwent lens transplantation at 30hpf.  Shown are a donor embryo (A, B), a host eye on with transplanted lens (C, D), and the control side of the host embryo (E, F).  Each eye is shown in both transmitted light and UV illumination as indicated.  Photographs were taken at 48 hpf.  The Q01^[9]^ transgenic line was used in this experiment.

## Discussion

Several steps require special attention during lens transplantations.

Sharpened needles:  It’s better to prepare several needles before carrying out lens transplantation, and the tip of the needles should be as thin as possible.  A thicker needle will tear more tissue because of its wider diameter, causing a failure in the eye to heal.
Arranging the embryos:  Right before transplantation you must position the embryos so they are lying on their sides.  When positioning, the 1.2% agarose can harden very quickly. To slow the hardening of agarose, the Petri dish can be preheated by letting it float in a 40°C water bath.
Before inserting the donor lens into the host eye, try to remove as much agarose around the host head as possible.  It’s easier to insert the donor lens that way.
Just after lens transplantation the embryos should be left embedded in the 1.2% agarose layer covered by a 0.2% agarose layer.  Adding embryo medium with antibiotics will promote wound healing in both donor and host embryos.  Wait 30-60 minutes before releasing embryos from agarose, and then transfer them into a 24-well plate with embryo medium.
